# Supplementary figures and images for: Time encoding migrates from prefrontal cortex to dorsal striatum during learning of a self-timed response duration task
Source: eLife. 2022 Sep 28;11:e65495. doi: 10.7554/eLife.65495 (PMC9519146; doi:10.7554/eLife.65495)

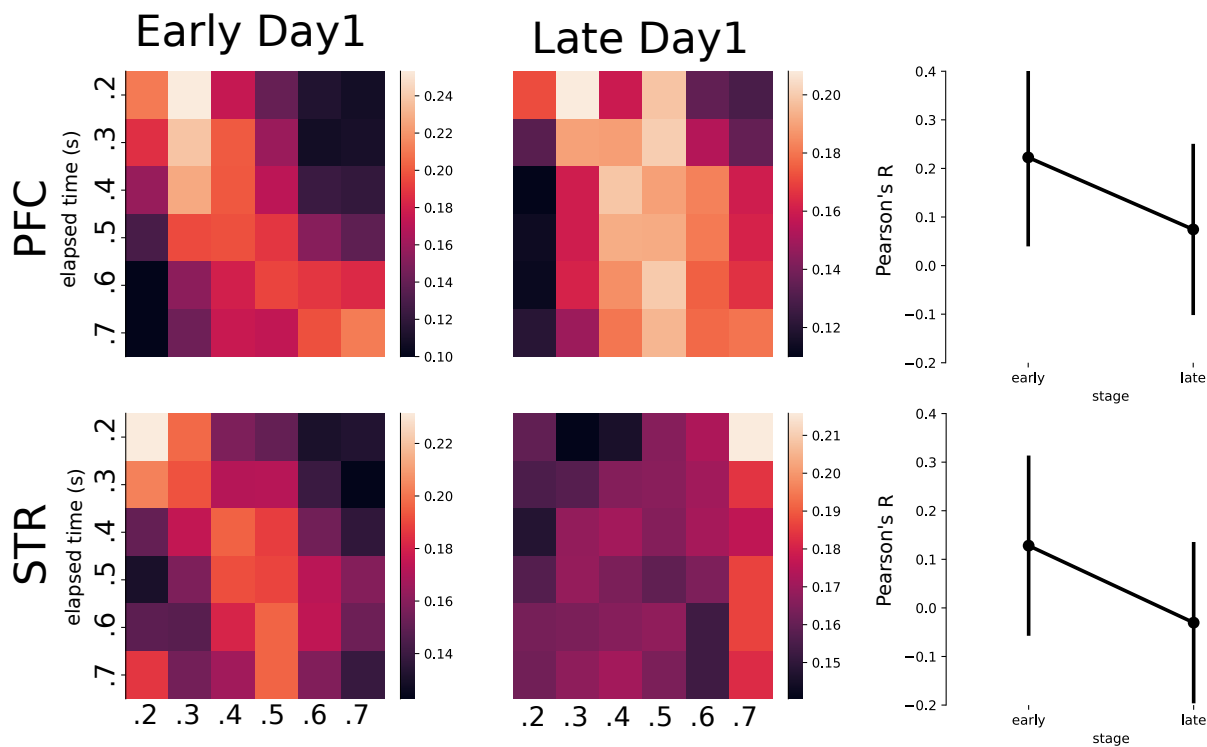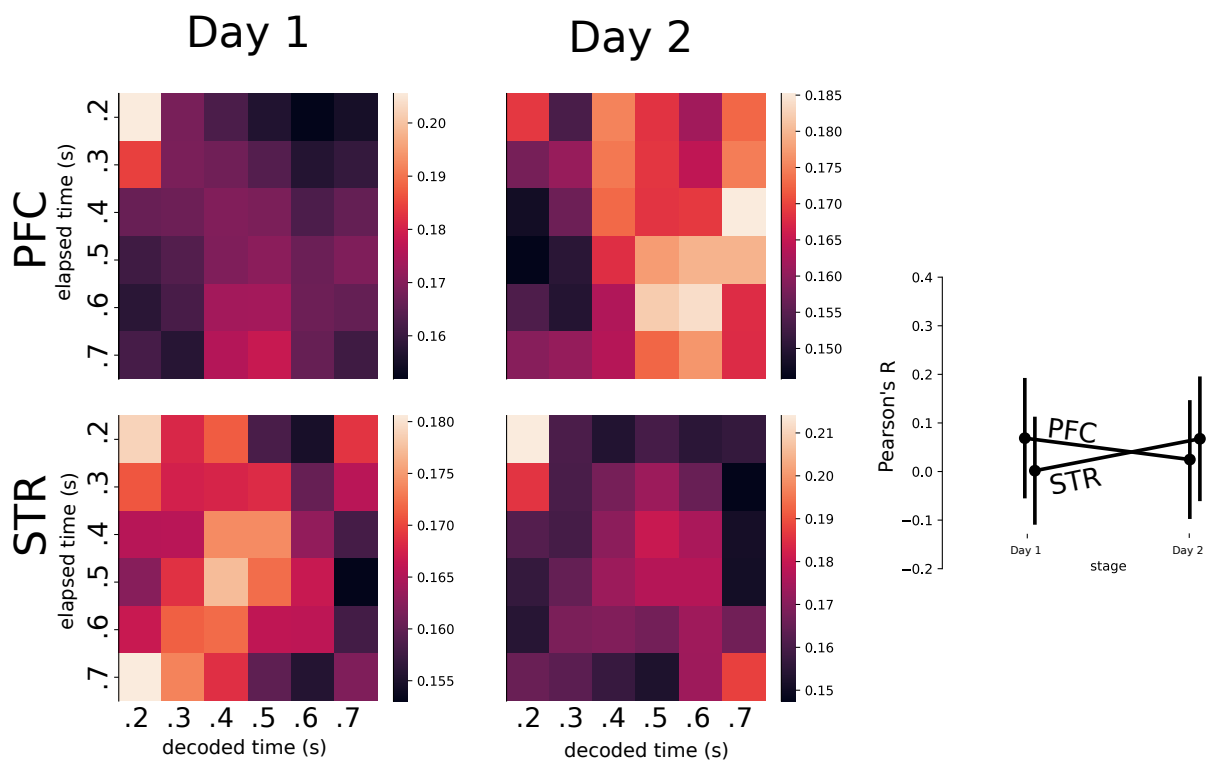

Supplement: Supplementary file 1. [file elife-65495-supp1.zip › supplementary-Fig_classifier_IncorrectTrials.pdf]
