# Supplementary material for: Time encoding migrates from prefrontal cortex to dorsal striatum during learning of a self-timed response duration task
Source: eLife. 2022 Sep 28;11:e65495. doi: 10.7554/eLife.65495 (PMC9519146; doi:10.7554/eLife.65495)
Supplement: Transparent reporting form [file elife-65495-transrepform1.pdf]

eLife's transparent reporting form

We encourage authors to provide detailed information *within their submission* to facilitate the interpretation and replication of experiments. Authors can upload supporting documentation to indicate the use of appropriate reporting guidelines for health-related research (see EQUATOR Network), life science research (see the BioSharing Information Resource), or the ARRIVE guidelines for reporting work involving animal research. Where applicable, authors should refer to any relevant reporting standards documents in this form.

If you have any questions, please consult our Journal Policies and/or contact us: editorial@elifesciences.org.

Sample-size estimation

- You should state whether an appropriate sample size was computed when the study was being designed
- You should state the statistical method of sample size computation and any required assumptions
- If no explicit power analysis was used, you should describe how you decided what sample (replicate) size (number) to use

Please outline where this information can be found within the submission (e.g., sections or figure legends), or explain why this information doesn't apply to your submission:

Replicates

We used a novel behavioral task in an electrophysiological and pharmacological context. Hence, there was no reference to the results' experimental error, which was essential for the sample size estimate. Therefore, the sample size estimation was based on previous studies using the same behavioral task, published in:
https://www.sciencedirect.com/science/article/abs/pii/S0376635719302086?via%3Dihub
We also gaged our sample sizes based on similar timing tasks:
Gouvêa, et al. (2015). Elife: 3 rats.
https://elifesciences.org/articles/11386#s4
Fan, D., Rossi, M. a, & Yin, H. H. J. Neuroscience (2012): 5 rats
https://www.jneurosci.org/content/32/16/5534.short

- • You should report how often each experiment was performed
- You should include a definition of biological versus technical replication
- The data obtained should be provided and sufficient information should be provided to indicate the number of independent biological and/or technical replicates
- If you encountered any outliers, you should describe how these were handled
- Criteria for exclusion/inclusion of data should be clearly stated
- High-throughput sequence data should be uploaded before submission, with a private link for reviewers provided (these are available from both GEO and ArrayExpress)

Please outline where this information can be found within the submission (e.g., sections or figure legends), or explain why this information doesn't apply to your submission:

We analyzed data from each animal separately for the behavioral and pharmacological results and, at the group level, compared them with statistical tests such as ANOVAs and t-tests. For the electrophysiological data, we combined neurons' activity from different animals, a common approach in the area. We clearly stated so in the Results section.

All the other inclusion criteria were described in the methods session.

Statistical reporting

- Statistical analysis methods should be described and justified
- Raw data should be presented in figures whenever informative to do so (typically when N per group is less than 10)
- For each experiment, you should identify the statistical tests used, exact values of N, definitions of center, methods of multiple test correction, and dispersion and precision measures (e.g., mean, median, SD, SEM, confidence intervals; and, for the major substantive results, a measure of effect size (e.g., Pearson's r, Cohen's d)
- Report exact p-values wherever possible alongside the summary statistics and 95% confidence intervals. These should be reported for all key questions and not only when the p-value is less than 0.05.

Please outline where this information can be found within the submission (e.g., sections or figure legends), or explain why this information doesn't apply to your submission:

(For large datasets, or papers with a very large number of statistical tests, you may upload a single table file with tests, Ns, etc., with reference to sections in the manuscript.)

The results session show the complete explanation of the statistical tests used along with the effect sizes.

Raw data is presented in all figures in which it was possible. Effect sizes are reported in all analyses, together with the statistical tests used.

Group allocation

- Indicate how samples were allocated into experimental groups (in the case of clinical studies, please specify allocation to treatment method); if randomization was used, please also state if restricted randomization was applied
- Indicate if masking was used during group allocation, data collection and/or data analysis

Please outline where this information can be found within the submission (e.g., sections or figure legends), or explain why this information doesn't apply to your submission:

eLife Sciences Publications, Ltd is a limited liability non-profit non-stock corporation incorporated in the State of Delaware, USA, with company number 5030732, and is registered in the UK with company number FC030576 and branch number BR015634 at the address 1st Floor, 24 Hills Road, Cambridge CB2 1JP | August 2014

Additional data files ("source data")

We used rats that were randomly assigned to groups as usual in animal research.

- • We encourage you to upload relevant additional data files, such as numerical data that are represented as a graph in a figure, or as a summary table
- Where provided, these should be in the most useful format, and they can be uploaded as "Source data" files linked to a main figure or table
- Include model definition files including the full list of parameters used
- Include code used for data analysis (e.g., R, MatLab)
- Avoid stating that data files are "available upon request"

Please indicate the figures or tables for which source data files have been provided:

All figures containing data were built from data files on osf website: https://osf.io/uh4s6/
